# Supplementary material for: Human Adipose Tissue-Derived Mesenchymal Stromal Cells Inhibit CD4+ T Cell Proliferation and Induce Regulatory T Cells as Well as CD127 Expression on CD4+CD25+ T Cells
Source: Cells. 2021 Jan 1;10(1):58. doi: 10.3390/cells10010058 (PMC7824667; doi:10.3390/cells10010058)
Supplement: Supplementary file 1 [file cells-10-00058-s001.pdf]

Supplementary Figures and Tables

Table S1. Percentages of CD4+CD25+ cells within the Live population.

**Culture condition**		**%CD4+CD25+ of Live**
Not stimulated PBMC	Direct	5.73 ± 1.15
	Transwell	7.85 ± 2.02
Not stimulated ASC cocultures	Direct	5.18 ± 0.33
	Transwell	5.84 ± 0.82
Stimulated PBMC	Direct	17.67 ± 2.17
	Transwell	13.92 ± 1.37
Stimulated ASC cocultures	Direct	22.34 ± 4.79
	Transwell	23.26 ± 4.33

Table S2. Percentages of CD4+CD25+ cells in the proliferated and not proliferated live fraction.

**Culture condition**		**%CD4+CD25+ in proliferated**	**%CD4+CD25+ in not proliferated**
Not stimulated PBMC	Direct	15.37 ± 5.75	4.08 ± 1.88
	Transwell	21.51 ± 8.92	5.26 ± 1.42
Not stimulated ASC cocultures	Direct	2.83 ± 2.5	5.25 ± 0.63
	Transwell	10.41 ± 4.77	6.36 ± 1.24
Stimulated PBMC	Direct	20.38 ± 3.16	15.01 ± 0.21
	Transwell	15.33 ± 1.21	14.30 ± 2.46
Stimulated ASC cocultures	Direct	35.41 ± 9.85	20.26 ± 4.02
	Transwell	40.23 ± 2.61	20.28 ± 1.67

Table S3. Percentages of CD4+CD25+CD127/FoxP3 subpopulations within the Live population referring to graph in Figure 5A′.

		**%CD4+CD25+CD127/FoxP3 in Live**		
**Culture condition**		**CD127-FoxP3+ (red)**	**CD127+FoxP3- (green)**	**CD127-FoxP3- (blu)**
Not stimulated PBMC	Direct	1.52 ± 0.24	0.85 ± 0.33	3.14 ± 0.71
	Transwell	1.79 ± 0.08	1.27 ± 0.51	7.59 ± 2.70
Not stimulated ASC cocultures	Direct	2.07 ± 1.81	1.89 ± 0.31	1.45 ± 0.26
	Transwell	2.83 ± 0.37	2.04 ± 0.43	2.26 ± 0.67
Stimulated PBMC	Direct	2.83 ± 0.37	3.86 ± 2.23	13.5 ± 0.92
	Transwell	2.24 ± 0.17	2.38 ± 1.34	9.24 ± 0.87
Stimulated ASC cocultures	Direct	3.63 ± 0.68	9.74 ± 2.40	7.84 ± 1.14
	Transwell	3.11 ± 0.66	10.43 ± 3.28	9.25 ± 0.71

Table S4. Percentage of CD4+CD25+CD127/FoxP3 in proliferated CD4+CD25+ cell fraction.

Culture condition		%CD4+CD25+CD127/FoxP3 in proliferated CD4+CD25+		
		**CD127-FoxP3+**	**CD127+FoxP3-**	**CD127-FoxP3-**
Not stimulated PBMC	Direct	8.99 ± 2.86	18.51 ± 4.40	71.70 ± 7.53
	Transwell	12.64 ± 3.92	13.19 ± 1.82	73.60 ± 5.34
Not stimulated ASC cocultures	Direct	32.77 ± 5.60	37.12 ± 3.43	28.69 ± 0.16
	Transwell	18.07 ± 9.78	29.66 ± 1.84	51.42 ± 11.24
Stimulated PBMC	Direct	13.55 ± 2.63	19.38 ± 10.29	66.80 ± 7.58
	Transwell	16.69 ± 2.92	19.67 ± 7.99	63.30 ± 6.81
Stimulated ASC cocultures	Direct	14.59 ± 2.86	44.03 ± 7.84	38.84 ± 8.96
	Transwell	8.51 ± 1.28	53.84 ± 6.01	35.45 ± 7.32

Table S5. Percentage of CD4+CD25+CD127/FoxP3 in not proliferated CD4+CD25+ cell fraction.

Culture condition		%CD4+CD25+CD127/FoxP3 in not proliferated CD4+CD25+		
		**CD127-FoxP3+**	**CD127+FoxP3-**	**CD127-FoxP3-**
Not stimulated PBMC	Direct	32.43 ± 2.50	13.63 ± 1.54	51.52 ± 3.76
	Transwell	32.61 ± 6.19	11.96 ± 0.31	53.45 ± 5.93
Not stimulated ASC cocultures	Direct	33.80 ± 5.83	35.95 ± 9.54	28.61 ± 5.72
	Transwell	30.53 ± 4.28	34.26 ± 2.59	32.61 ± 3.52
Stimulated PBMC	Direct	19.73 ± 4.81	19.15 ± 6.02	59.65 ± 10.38
	Transwell	16.97 ± 0.77	20.31 ± 2.73	61.32 ± 2.69
Stimulated ASC cocultures	Direct	15.33 ± 3.51	46.23 ± 1.94	36.37 ± 2.46
	Transwell	15.56 ± 2.63	42.80 ± 4.60	39.08 ± 5.01
